# Isolation and Characterization of Adenoviruses Persistently Shed from the Gastrointestinal Tract of Non-Human Primates

**DOI:** 10.1371/journal.ppat.1000503

**Published:** 2009-07-03

**Authors:** Soumitra Roy, Luk H. Vandenberghe, Sergey Kryazhimskiy, Rebecca Grant, Roberto Calcedo, Xin Yuan, Martin Keough, Arbans Sandhu, Qiang Wang, C. Angelica Medina-Jaszek, Joshua B. Plotkin, James M. Wilson

**Affiliations:** 1 Department of Pathology and Laboratory Medicine, University of Pennsylvania, Philadelphia, Pennsylvania, United States of America; 2 Department of Biology, University of Pennsylvania, Philadelphia, Pennsylvania, United States of America; 3 Vaccine Research Institute, Guangzhou, China; Cornell University, United States of America

## Abstract

Adenoviruses are important human pathogens that have been developed as vectors for gene therapies and genetic vaccines. Previous studies indicated that human infections with adenoviruses are self-limiting in immunocompetent hosts with evidence of some persistence in adenoid tissue. We sought to better understand the natural history of adenovirus infections in various non-human primates and discovered that healthy populations of great apes (chimpanzees, bonobos, gorillas, and orangutans) and macaques shed substantial quantities of infectious adenoviruses in stool. Shedding in stools from asymptomatic humans was found to be much less frequent, comparable to frequencies reported before. We purified and fully sequenced 30 novel adenoviruses from apes and 3 novel adenoviruses from macaques. Analyses of the new ape adenovirus sequences (as well as the 4 chimpanzee adenovirus sequences we have previously reported) together with 22 complete adenovirus genomes available from GenBank revealed that (a) the ape adenoviruses could clearly be classified into species corresponding to human adenovirus species B, C, and E, (b) there was evidence for intraspecies recombination between adenoviruses, and (c) the high degree of phylogenetic relatedness of adenoviruses across their various primate hosts provided evidence for cross species transmission events to have occurred in the natural history of B and E viruses. The high degree of asymptomatic shedding of live adenovirus in non-human primates and evidence for zoonotic transmissions warrants caution for primate handling and housing. Furthermore, the presence of persistent and/or latent adenovirus infections in the gut should be considered in the design and interpretation of human and non-human primate studies with adenovirus vectors.

## Author Summary

Adenoviruses were originally discovered as a causative agent for upper respiratory infections, especially in children. They are now recognized as being responsible for disease in several other settings, e.g., as the cause of infectious diarrheas or of kerato-conjunctivitis. In the 1960s, chimpanzees were found to shed adenoviruses in their stools. We now find that normal great apes (chimpanzees, gorillas, and orangutans) and monkeys frequently shed adenoviruses in their stools, corroborating the older finding. In contrast, data from humans have showed that adenoviral shedding in stools is fairly uncommon, being restricted to patients recovering from adenoviral infections, although adenoviruses can frequently be recovered from lymphoid tissue such as the adenoids from normal people. In the course of these investigations we have purified and characterized 30 different adenoviruses from great apes by genomic sequencing. We have compared the gene sequences of these adenoviruses to 22 human adenoviruses whose complete genome sequences are available. Even though human adenoviruses are not found in apes and *vice versa*, based on these sequence data we propose that it is possible that cross-species transmission events may have contributed to the natural evolution of adenoviruses found to infect humans today.

## Introduction

Adenoviruses were first discovered in the 1950s as outgrowths from human adenoid and tonsil explants. They naturally infect many different vertebrates including humans. Isolates were originally found to differ in their ability to agglutinate rat and rhesus erythrocytes and these properties were used to classify adenoviruses into four subgroups [1]. Subsequently, other prominent attributes of adenoviruses, e.g., the ability to induce tumors in rodents, the molecular masses of capsid proteins as determined by electrophoresis [2] and GC content of the genome [3] were used to augment the four initial subgroups to the six (subgroups A to F, currently referred to as species HAdV-A through HAdV-F) that are commonly accepted today. As complete sequences of the human adenoviruses have become available and phylogenic analyses of the component proteins have become possible, the original classifications have been confirmed to be reasonably valid although some reservation has been expressed regarding the justification of assigning a separate subgroup (E) to its only defined human adenovirus member, HAdV-4 [4].

Infections with HAdV-C are a common cause of pediatric upper respiratory tract infections worldwide; later in life HAdV-B has been found to the cause of epidemics in adults. Both HAdV-B and HAdV-E have been found to the etiologic agents for infections in the setting of military boot camps [5]. Surveys for the prevalence of neutralizing antibodies have been conducted in order to determine the probable success of recombinant adenoviral vaccines [6], where the prevalence of antibodies to HAdV-C has found to be high in Europe, USA and Africa [7]. In contrast, the prevalence of antibodies to HAdV-B has been found to be comparatively lower in a European cohort [8]. HAdV-F is frequently implicated as a cause of infectious diarrheas worldwide [9]–[14], but other adenovirus serotypes were also frequently co-isolated under these conditions.

The molecular biology of primate adenoviruses has been defined in substantial detail [15]. Less is known about the natural history of naturally occurring adenovirus infections. Recombinant forms of human adenoviruses have been aggressively pursued as vectors for human gene therapy and genetic vaccines [16].

Natural history studies of large cohorts of families in Seattle [17] and Long Island [18] showed that clinical sequelae of adenovirus infections and recovery of virus in immune competent humans are self–limited, although prolonged shedding was observed in stool from a subset of individuals. An early clue to the biology of adenovirus infections in great apes was provided by Hillis in 1963 in a study which attempted to isolate the cause of transmissible hepatitis in chimpanzees [19]. He recovered from stool of 25/87 chimpanzees an uncharacterized cytopathic virus that grew *in vitro* but was not related to hepatitis. He concluded that “there are a number of enterically excreted viruses in the chimpanzee, some or all of which may be part of the animal's ‘normal flora’ ”.

## Results

To better understand the natural history of adenovirus infections in primates, we investigated virus persistence and latency in species of the Hominidae and the Cercopithecidae family. Samples were acquired from several great ape species: chimpanzee (*Pan troglodytes*), bonobo (*Pan paniscus*), orangutan (*Pongo pygmaeus*) and gorilla (*Gorilla gorilla*) as well as normal humans. Two old world monkey species, rhesus and cynomolgus macaques (*Macaca mulatta* and *Macaca fascicularis*, respectively), were also studied since they are commonly used to model human diseases and to evaluate efficacy and safety of adenoviral vectors for gene therapy and genetic vaccines. We reasoned that in these primates the gut may serve as a site of persistence, and that evidence of this phenomenon could be obtained from stool extracts by culture on cells permissive for adenoviral replication or through a sensitive PCR assay for direct detection of adenoviral DNA (Table 1).

**Table 1:** Recovery of adenoviruses from primate stools.

	CPE+		PCR+	
**Chimpanzees**				
**New Iberia**	29/82	(35%)	30/30	(100%)
**MD Anderson**	36/142	(25%)	29/32	(91%)
**Zoos**	1/11	(9%)	4/11	(36%)
**DRC**		ND	9/24	(42%)
**Cameroon**		ND	18/43	(38%)
**Bonobos**				
**Zoos**	5/13	(38%)	6/13	(46%)
**Gorillas**				
**Zoos**	11/45	(24%)	18/45	(40%)
**Wild/Africa**	0/6	(0%)	3/6	(50%)
**Orangutans**				
**Zoos**	0/27	(0%)	17/27	(63%)
**Humans**				
**USA adults**		ND	0/18	(0%)
**China adults**	10/73	(14%)	2/73	(3%)
**China children**	6/41	(15%)	3/41	(7%)
**Macaques**				
**Oregon – rhesus**	1/100	(1%)	6/48	(13%)
**Covance – rhesus**	16/100	(16%)	32/48	(67%)
**GTP – rhesus**	0/6	(0%)	6/6	(100%)
**GTP – cyno**	3/42	(7%)	37/42	(88%)

Samples were analyzed by culturing virus on relevant indicator cells or were subjected to a nested PCR for a highly conserved region of the adenoviral DNA polymerase gene. All samples that showed cytopathic effect when exposed to permissive cells were analyzed by a hexon-specific PCR to confirm the presence of adenovirus. CPE+ indicates the number rescued/total number analyzed (percentage recovery). PCR+ shows the results of the nested PCR with the number of PCR positive samples over the total number of samples tested as a denominator. (ND: not determined).

Non-human primate stool specimens were retrieved from animals in three distinct living conditions. First, stool from chimpanzees and gorillas that live in the wild in several locations in Africa were assayed for the presence of viral genomes by PCR. Attempts to isolate live, infectious virus from the PCR positive stool samples were unsuccessful (Table 1) and highlighted the technical limitations (numbers, quality, condition and preservation) with specimen from animals in the African wild. In order to test whether PCR positive stools also harbored live virus, we obtained more recent stool samples from several primate facilities in the United States that housed chimpanzees (New Iberia Research Center of the University of Louisiana at Lafayette, and the Michale E. Keeling Center for Comparative Medicine and Research, University of Texas M. D. Anderson Cancer Center) and macaques (Covance Research Products Inc., Oregon National Primate Research Center and the Non-human Primate Research Program at the University of Pennsylvania). Thirdly, we addressed the question whether the findings from chimpanzees and macaques extended to other primates by assaying fecal specimens from several great ape species that lived in captivity in ten different zoos in the United States.

**Figure 1:**
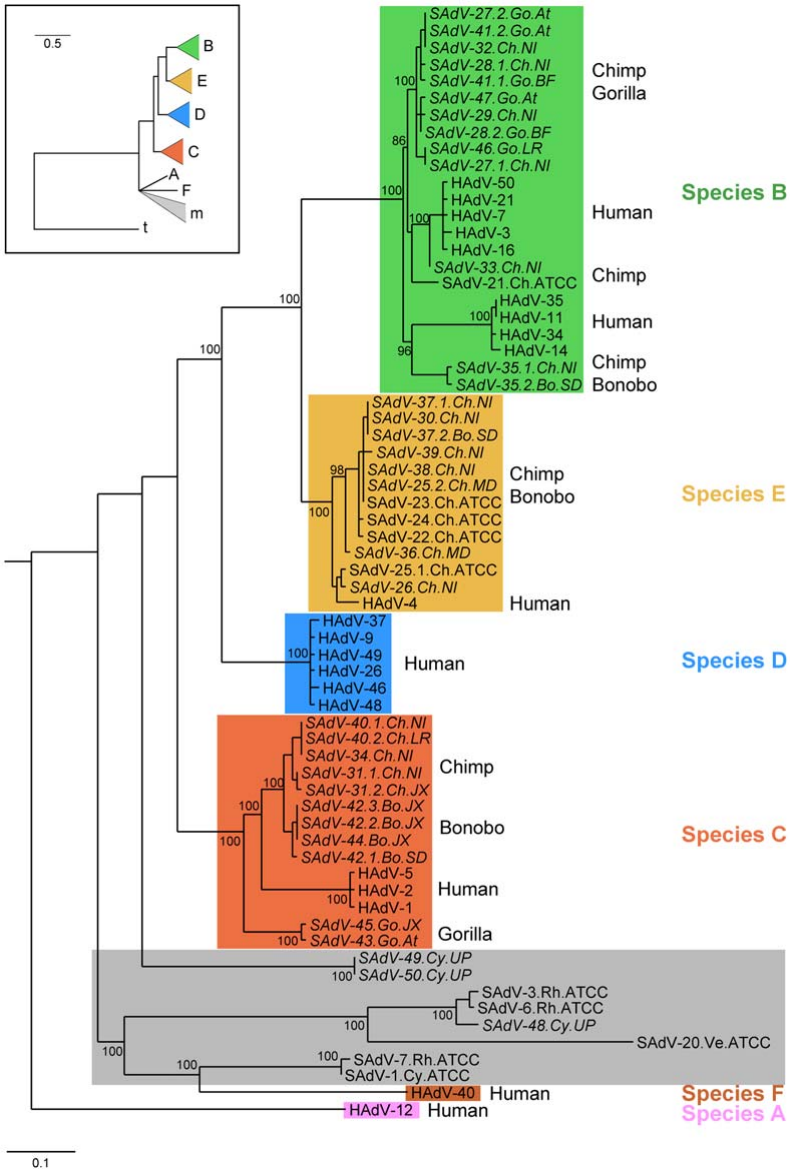
Phylogenetic tree of adenoviruses that infect primates. The tree was reconstructed from an alignment of the polymerase gene, using maximum likelihood under the HKY85 model of substitutions, as described in Materials and Methods. The names of simian isolates include the serotype nomenclature, the animal species of isolation (Hu: human, Ch: chimpanzee, Bo: bonobo, Go: gorilla, Cy: cynomolgus macaque, Rh: rhesus macaque), and the source of adenoviral isolation (ATCC: American Tissue Type Collection, JX: Jacksonville zoo, NI: New Iberia Research Center, MD: MD Anderson, SD: San Diego zoo, At: Atlanta zoo, LR: Little Rock zoo, BF: Buffalo zoo). Isolates that have a closely related hexon structure are referred to as variants of the same serotype (“.1” or “.2”). Names of novel sequences obtained in this study are shown in italics. Colors indicate the six species of viruses that infect higher primates (A, B, C, D, E, and F); grey is used to indicate viruses isolated from monkeys. The inset (upper left) shows the same tree with the inclusion of a tree-shrew isolate as an outgroup, after collapsing poorly supported bifurcations. Bootstrap values less than 80% or close to terminal leaves are suppressed.

Chimpanzee stool samples collected from natural habitats in Cameroon and the Democratic Republic of Congo (DRC) were made available to us for analysis. DNA isolated from these samples has been used for simian foamy virus studies [20]. These samples underwent a sensitive nested PCR that was designed to directly detect adenovirus genomes using oligonucleotides complementary to a conserved region of the DNA polymerase gene (*pol*). Adenovirus DNA could be detected in 40% of the stool samples from chimpanzees that exist in the wild (Table 1). Cloning and sequencing of these PCR products identified twenty-four that were species E and three that were species B (data not shown). In this conserved region, species E viruses closely clustered with SAdV-25.1, 26 and 39 whereas species B isolates where mostly similar to SAdV-35.1 and SAdV-35.2 (Figure 1). We also obtained stool from mountain gorillas in the Virunga Massif mountain range in Rwanda and detected adenovirus in 3/6 samples by *pol* PCR. Stool extracts were also filtered and evaluated for their ability to induce cytopathic effects (CPE) on the human epithelial A549 cell line. Every attempt to grow out live adenovirus on A549 cells was unsuccessful. We were unable to determine whether this failure was due to the technical limitations of sample collection or preservation, the sensitivity of the cellular detection assay or the absence of functional viral particles. Analysis of samples acquired under more controlled conditions in the USA permitted us to address these important questions.

Samples received from USA facilities underwent two parallel assays. A PCR based assay was designed to detect adenoviral DNA at high sensitivity and specificity for adenovirus using primers corresponding to a conserved region of *pol* as described above. A second assay aimed to recover replication competent live adenovirus. The substrates for both assays were the stool droppings that were re-suspended and filtered. The infectivity of these adenoviral genomes in great ape fecal specimens was tested in conditions amenable to replication of human adenoviruses by inoculating A549 cells and evaluating the cultures for CPE. Macaque stool was processed identically but cultured on a more permissive cell line for the growth of macaque adenoviruses than A549 i.e. an African green monkey epithelial kidney cell line named BS-C-1. In a subsequent step, CPE positive samples underwent a PCR assay in which a portion of the hexon gene that spans hypervariable regions 1 to 6 was amplified [21]. Cloning and sequencing of these PCR fragments provided confirmation of the presence of adenovirus and the relatedness of the isolate to other known adenoviruses. Based on this information, selected samples were further amplified in culture and complete genomic sequence was determined.

Of the 568 non-human primate stool samples from zoos and primate facilities, 18% tested were found to be positive for adenovirus by the CPE detection assay (Table 1). This assay provided evidence of shedding of infectious adenovirus for all species at the time of sampling except for samples from orangutans which failed to yield infectious virus under the culture condition employed. Shedding was observed at similar frequencies (22–38%) among chimpanzees, bonobos and gorillas. The macaque specimens yielded a substantially lower incidence of CPE (8%).

Of the great ape isolates, all amplified hexon fragments were sequenced and classified phylogenetically according to adenoviral species. The 65 PCR positive chimpanzee samples from primate facilities yielded 15 species B, 28 species C and 23 species E that clustered in 16 clades based on sequence similarity (>95% identity). Fifteen representative isolates of the 16 clades were further amplified and the viral DNA purified and completely sequenced (Figure 1). Phylogenies of representative open reading frames (ORFs) of several portions of the genomes largely reflected the earlier classification based on partial hexon sequence with a few exceptions (Figure 1 and Figures S1, S2, S3, S4, S5, S6 and S7). For example, the species E strain SAdV-25.2 encodes a hexon highly similar to a previous isolate SAdV25.1. Interestingly, the phylogeny of these two viruses diverges in most other ORFs studied, suggestive of recombination playing a role in the evolutionary history of these isolates. Adenoviruses isolated from zoos belonged mostly to species B (gorilla: 5, bonobo: 2) and species C (chimpanzee: 2, gorilla: 2, bonobo: 4) with only one bonobo virus in species E. All zoo isolates were successfully grown up and fully sequenced. Human species B adenoviruses that have previously been sub-classified into B:1 (HAdV-3, HAdV-7, and HAdV-21) and B:2 (HAdV-11, HAdV-34, and HAdV-35) sub-clades based on restriction enzyme digestions patterns are seen to cluster separately and are seen to have corresponding counterparts in the ape adenovirus isolates. SAdV-27, SAdV-28, SAdV-29, SAdV-32, SAdV-27, SAdV-41, SAdV-46 and SAdV-47 cluster with the human B:1 isolates whereas SAdV-21, SAdV-33 and SAdV-35 are more closely related to human B:2 isolates.

A subset of stool samples from each captive population of non-human primates (experimental facilities and zoos) was analyzed by direct *pol* PCR since we hypothesized that the CPE assay to detect adenovirus in stool likely underestimates the actual frequency of shedding for two reasons: samples were not freshly isolated, and the cell line used for recovery was not necessarily optimal for the respective adenoviruses. Indeed, in each population studied the detection of adenovirus by PCR was more sensitive than that based on culture except for humans where sample collection was carried out under more controlled conditions followed by culturing on a human cell line (Table 1). Adenovirus sequence was detected in stools of over 90% of chimpanzees from the primate facilities, 36% of chimpanzees from zoos, 46% of bonobos from zoos, and 40% of gorillas from zoos. Orangutan-derived stool yielded positive PCR signals in 63% of samples despite that fact none of the samples contained viruses that could be rescued on A549 cells. In macaques, the increased sensitivity of the PCR diagnostic assay yielded a higher prevalence in every population studied with overall evidence of adenoviral shedding in 56% of macaques.

A number of previous studies of adenovirus infections in humans have attempted to isolate virus from stool samples. Viruses of the species F group cause infectious diarrhea and are often isolated from stools of symptomatic individuals [9]–[14],[22]. The aforementioned natural history studies demonstrated some examples of prolonged shedding in stool after resolution of respiratory infections [17],[18]. Screening of stool samples from asymptomatic normal subjects using a variety of techniques has demonstrated recovery of adenovirus in 2 to 3% of individuals [23],[24]. Our rate of recovery of adenovirus from stools of normal humans (i.e., no concurrent infectious illness) was similar to those previously reported, using the same technique used to isolate and detect adenovirus from great apes (Table 1). Samples were obtained from several groups who at the time of collection were not suffering from an infectious illness including adults from USA (N = 18) and adults (N = 73) and children (N = 41) from China. Samples from USA were negative by PCR. Samples from China showed detectable CPE in 14% of adult and children samples, most of which were confirmed by PCR to be due to adenovirus; direct analysis of stools by nested primer PCR detected adenovirus sequence in 3% of adults and 7% of children in China. Most adenoviruses recovered by CPE and direct PCR analysis were characterized with respect to sequence across hexon and polymerase indicating the presence of species B (HAdV-7) and C (HAdV-2 and 5) viruses as was the case with the great apes; however, species A (HAdV-12), D (HAdV-48 and HAdV-26) and F (HAdV-41) adenoviruses were also detected.

A total of 30 apparently novel adenoviral genomes (out of 33 that were sequenced) from the great ape samples described above were sequenced in their entirety and subjected to phylogenetic analysis, shown in Figure 1 and Figures S1, S2, S3, S4, S5, S6 and S7. Our analysis also included three viruses we isolated from cynomolgus macaques (which were completely sequenced), along with reference sequences from previously published human and simian isolates as well as a tree shrew-derived isolate [25]–[27]. Standardized nomenclature is used for the adenoviruses, including the prefix H or S indicating isolation from a human or simian source. There was no discernible difference in the genomic structure of the adenoviruses isolated from great apes and macaques compared to those isolated from humans, except for variations in the E3 region [28].

Using maximum likelihood, we constructed phylogenetic trees based on each of the following gene sequences: DNA polymerase (Figure 1), E1a (13S mRNA analog encoded, Figure S1), pre-terminal protein (pTP, Figure S2), hexon (Figure S3), penton base (Figure S4), protease (Figure S5), ssDNA-binding protein (DBP, Figure S6), and fiber (Figure S7). Overall, these trees were concordant with several notable exceptions. For one, species D branching in E1a and hexon is discordant as compared to the other proteins studied. Also, the phylogeny of the highly divergent fiber protein is distinctive and possibly reflective of differences in receptor usage (CD46 for species B as opposed to Coxsackie Adenovirus Receptor for the others; species D fibers are known to additionally bind sialic acid). The phylogeny of species A and F, and their relationship to the macaque isolates is highly discordant for the different proteins. The cynomolgus isolates, SAdV-49 and SAdV-50, for example, are closely associated with species A in E1a but are either closer to species F or are divergent for the other proteins. Internally within clades, discordance is also sometimes noted in species B and E and may be indicative of intra-species recombination. We used the polymerase tree (Figure 1) as a reference phylogeny throughout the manuscript. Overall, the phylogeny of the viral sequences reflects the host species from which the viruses were isolated. All viruses from great apes and humans except species A and F viruses form a monophyletic group, whereas adenoviruses infecting tree shrew, macaques, and bovines (not shown) fall outside this group [29]. The macaque-derived viruses SAdV-49 and SAdV-50 were positioned in a previously uncharacterized clade, yet certain genes have close resemblance to the same genes in species A and F adenoviruses. SAdV-48, another macaque-derived virus that we isolated, was more closely related to SAdV-3 and SAdV-6, both previously isolated from rhesus macaques (see Figure 1 and Figures S1, S2, S3, S4, S5, S6 and S7). The viruses isolated from great apes segregated into distinct clades within the larger groups of species B, C and E viruses. Human-derived viruses formed separate sub-clades from ape-derived viruses in each of these viral species. Reconstructed phylogenies consistently place D viruses in a monophyletic group with B, C, and E viruses but we did not identify any simian viruses belonging to species D. The phylogenies consistently place human A and F viruses in distinct and somewhat distant clades from all other human viruses. In fact, the A and F viruses are more closely related to macaque strains than they are to all other human viruses.

In addition to phylogenetic reconstructions, we also estimated protein evolutionary rates for the eight genes described above, by maximum likelihood. In this analysis nucleotide substitutions that change the amino acid sequence (dN) are compared to nucleotide substitutions that are silent (dS). All genes showed a predominance of selection against protein-coding substitutions (dN/dS<1 in all cases, mean dN/dS = 0.134) which is consistent with the corresponding proteins having structural constraints and playing an important role in the fitness of the virus. We also computed branch-specific dN/dS values for viruses that infect human versus non-human hosts. We found a significant elevation of dN/dS (p<0.0001) for the fiber gene along human-host lineages (dN/dS = 0.270) as compared non-human-host lineages (dN/dS = 0.208). This result is consistent with increased pressure on the evolution of the fiber proteins in humans relative to primates (or relaxed negative selection), which may be mediated by humoral immunity since the fiber is exposed on the surface of the virus.

**Figure 2:**
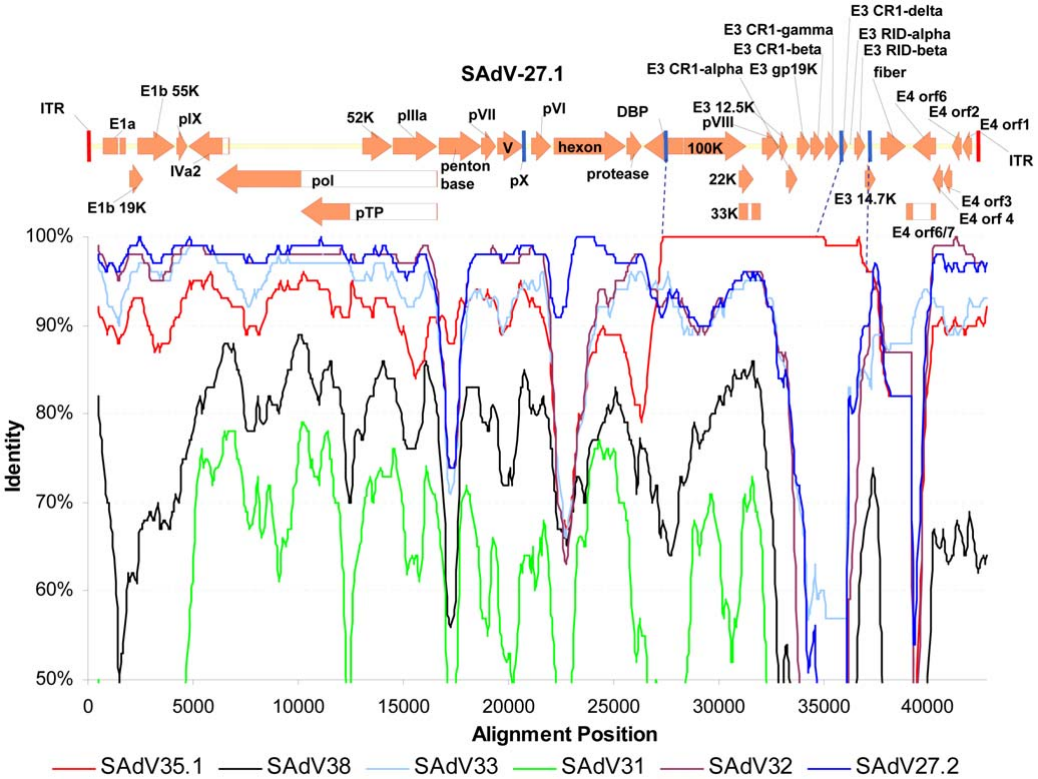
Evidence for intra-species B recombination within the New Iberia chimpanzee colony. A Simplot analysis of New Iberia species B adenoviral isolates aligned with a schematic annotated genomic structure of the SAdV-27.1 showing evidence of an incidence of recombination between viral isolates from the same compound. The analysis demonstrates homology on the left genomic end extending up to the DNA Binding Protein (DBP) open reading frame with various species B isolates from within one subspecies (represented by SAdV-32) from the New Iberia chimpanzee colony, followed by an 100K, 22K, 33K and partial E3 gene region of near complete identity to the structurally distinct New Iberia isolate SAdV-35.1 of a distinct subspecies within species B. To the right of this region of identity the homology transitions to the fiber region first to B isolate SAdV-33 and subsequently to SAdV-32 again. Further evidence is provided by SAdV-27.2, isolated from a gorilla in a different geographical location (Atlanta zoo). This genome is closely related to SAdV-27.1 and is almost identical in hexon but did not undergo the presumed recombination event between DBP and E3. The left and extreme right ends of the SADV-27.1 genome demonstrate close but not complete identity to SAdV-32 indicating a close relationship to the putative parental adenovirus of this event of homoplasy. The left junction of the recombination event is a clearly defined junction centrally in the DNA binding protein as indicated by the dotted blue line. The right end junction was less well-defined due to a region of lack of complete identity in the fiber-proximal part of the coding region of the E3 gene family. This region is indicated by the dotted blue lines in the sequence. The more distantly related species B isolate SAdV-35, species C isolate SAdV-31 and species E isolate SAdV-38 (all isolated from the New Iberia chimpanzee colony) are provided in the analysis as reference.

The analyses described above highlighted discordant phylogenies suggestive of recombination. To address this question more specifically, we subjected sequence datasets from every independent source of isolation to a computational analysis designed to detect recombination events. We performed a sliding window analysis of identity and bootstrap values of separate isolates in relation to the query sequence across the genome. Figure 2 illustrates a sliding window analysis of identity on SAdV-27.1, the most obvious example of recombination among viruses from the same location. SAdV-27.1, which is an isolate from the New Iberia chimpanzee facility, was queried against a number of related genomes including 3 other chimpanzee clade B isolates from the same combine (SAdV-32, SAdV-33, SAdV-35.1), a gorilla isolate with a highly identical hexon sequence (SAdV-27.2), and several reference isolates that served as outgroups (SAdV-31 and SAdV-38). This analysis revealed close identity at the left and extreme right ends between two species B.1 viruses of the same chimpanzee facility (SAdV-32 and SAdV-33) and the gorilla-derived SAdV27.2. Remarkably, the region starting on the left within the DBP open reading frame and extending to the end of the E3 locus was almost identical to the species B.2 isolate, SAdV-35.1, from the same facility. These findings identify SAdV-27.1 as a hybrid B.1-B.2 virus that was generated following a recombination event that occurred in the New Iberia compound between SAdV-35.1 or a very similar virus and a B.1 strain more distantly related to SAdV-32 and SAdV-33. Given that SAdV-35.1 and SAdV27.1 were received over 1 month apart, isolated from distinct combines at different dates and in distinct experiments, we excluded this recombination event as an *in vitro* culture artifact. These data illustrate the potential for intra-species recombination of adenoviruses and identify the first hybrid B.1–B.2 adenovirus.

## Discussion

We have detected a high prevalence of adenoviruses in the stools of apparently healthy animals of several great ape and macaque species. The high rate of shedding was surprising and suggests that adenoviruses are capable of establishing chronic infections following an initial exposure that may or may not be clinically relevant. The presence of adenovirus in stools from wild chimpanzees and gorillas across several countries in central Africa confirmed that this was not an artifact of animals being maintained in captivity. Our studies cannot rule out an alternative explanation in which animals are repeatedly infected although these infections would have to be prolonged and frequent to explain the high prevalence. There is substantially less shedding of virus in human stool although adenovirus genomes are highly prevalent and abundant in gut associated lymphoid tissue (data not shown). Analyses of host responses to these persistent/latent infections suggests that better control of the chronic infection in humans may be due to a more effective T cell response to the virus [28]. Our studies also suggest that the gut may serve as a source of the disseminated and often lethal adenovirus infections observed in human patients who are immune suppressed [30],[31].

Sequencing the complete genomes of the 33 novel adenoviruses (30 different sequences were found) that we have isolated from chimpanzees and gorillas has allowed us to examine the phylogenetic relationships among hominid adenoviruses. Overall, we found that the viruses isolated from great apes fall into the three species groups (B, C, and E) previously believed to be primarily of human origin. Generally, most DNA viruses are thought to have evolved with their host in a mechanism referred to as co-evolution [32] in which the virus was a pathogen of a common ancestor. Subsequent viral speciation is then expected to mirror primate speciation. In fact, the primate species C adenovirus phylogeny in this study is largely consistent with this hypothesis. However, several elements of the data, particularly in adenoviral species B and E, suggest that co-evolution alone may not fully explain the observed diversity and indicate a contribution of cross-species transmission to the primate adenoviral evolutionary history. Indeed, within species B and species D, the distances between certain human and ape adenovirus proteins are smaller than predicted by co-evolution, which is supported by high bootstrap values. In addition, partial hexon sequence of chimpanzee species C isolates that we were unable to grow out to obtain a full length sequence were found to cluster tightly with gorilla isolates indicating a possible cross-species transmission event in this species C subclade (data not shown). In addition, under the co-evolution hypothesis, one would expect the evolutionary distances between adenovirus species from humans to be similar to those from apes, assuming similar selective pressures. On the contrary, we observe that these distances are indeed very variable. For example, in clade B, SAdV-33 is only 0.018 substitutions/site removed from the human clade B.1 whereas the SAdV-35 viruses are almost 10 times more removed from the human clade B.2 viruses (0.170 substitutions/site). We may interpret these data as either evidence for dramatic differences in selective pressures or an indication of at least one, possibly two, independent cross-species transmission events at distinct points in time. When one applies conservative molecular clock estimates for DNA viruses [33], the time estimates of the divergence between close human and ape viruses in species B and E are also inconsistent with the date of the human-chimpanzee divergence.

Thus, we have demonstrated that cross-species transmission can be invoked to explain some of the phylogenetic relationships among human- and ape-infecting adenovirus isolates within the B and E species. We speculate that this transmission occurred from great apes to humans. The opposite direction of transmission cannot be ruled out, but is unlikely given the epidemiological evidence in favor of great apes as the natural host species.

Several limitations to the data prevent us from delineating a definitive model of evolutionary history of primate adenoviruses. First, the inability to isolate adenoviruses from certain clades or host species of non-human hosts may indicate either their extinction in that particular host or a failure to detect the virus to date due to limitations in sampling or assaying. These limitations make it extremely difficult to perform an exhaustive study and nor can the data be interpreted as such. This study does, however, provide the most extensive survey of non-human primate adenoviruses to date. It is clear that more extensive studies in primates, particularly those in great apes in the wild are warranted. All full-length adenoviral genomes presented here were isolated from animals in captivity yet limited *pol* sequence data from gorillas and chimpanzees in the wild provide preliminary evidence to confirm the fecal shedding of species B and E viruses. As stated in Results, the samples from the wild harbored predominantly species E sequences as determined by PCR amplification of the polymerase gene (24 belonging to species E and 3 to species B) whereas we were able to detect considerable numbers of species C adenoviruses (in addition to species E and B) in captivity. It can be speculated that the prevalence of the different adenovirus species in the gut of captive apes may be skewed as a result of captivity, but clearly more data including virus isolation in the wild is necessary in order to definitively confirm this observation.

The high prevalence of adenovirus shedding in great apes and their close relation to existing human adenoviruses suggest a potential risk of ape-to-human transmission. This risk will likely be higher where contacts are more prevalent, such as with captive and habituated animals. The limited available sero-epidemiological data for chimpanzee species B and E adenoviruses suggests this may be the case in areas home to common chimpanzee species [34]. In addition, the possibility that the adenoviruses that were isolated from animals in combines and zoos were an artifact of their captivity needs to be addressed. In fact these concerns were aired following the isolation of SAdV-22, SAdV-23, SAdV-24, SAdV-25 [27]. In that study, none of the animal handlers who were exposed to the chimpanzees were found to harbor antibodies to the chimpanzee adenoviruses. More recently, serum samples from 50 zoo workers were tested for the presence of neutralizing antibodies to SAdV-E (SAdV-23 and SAdV-25) and SAdV-B (SAdV-21), and none was found [35]. Although macaques shed adenovirus at high levels, transmission to humans seems unlikely since the macaque viruses are quite distinct from most known human adenoviruses. It is interesting however that an adenovirus similar to the macaque viruses was isolated from a patient with gastroenteritis [36].

These studies should also be considered in the context of applications of recombinant adenoviruses as vectors for human gene therapy and genetic vaccines. Chronic infections and latency of viruses and the associated host immune responses may confound the application of vectors that are related to these endogenous viruses.

## Materials and Methods

### Ethics statement

All animals were handled in strict accordance with good animal practice as defined by the University of Pennsylvania Institutional Animal Care and Use Committee, and all animal work was approved by this committee.

### Isolation of novel adenoviruses from stool

Stool samples were collected at the facilities that house the animals and were suspended in Hanks' Balanced Salt Solution (HBSS), and sent to University of Pennsylvania on ice. The particulates were removed by centrifugation, and the supernatant was sterile filtered through 0.2 µm syringe filters. 100 µl of each filtered sample was inoculated into A549 cells grown in Ham's F12 medium with 10% fetal bovine serum (FBS), 1% Penn-Strep and 50 µg/ml gentamicin. After about 1 to 2 weeks in culture, visual CPE was obvious in cell cultures with several of the inoculates. The presence of adenoviruses in the cultures was confirmed by PCR amplification of an internal 1.9 kb of the hexon – the region encompassing the hypervariable regions and that is predominantly responsible for conferring serotype specificity. The primer pair that was utilized for PCR was CAGGATGCTTCGGAGTACCTGAG and TTGGCNGGDATDGGGTAVAGCATGTT. The sequence obtained from this region was used to make an initial determination of adenoviral species and novelty of the serotype. Adenoviral isolates were plaque purified on A549 cells, propagated to high titer and purified on cesium chloride gradients using standard procedures. Viral DNAs obtained from purified virus preparations were completely sequenced (Qiagen Genomics Services, Hilden, Germany). In addition, a sensitive nested PCR specific for the DNA polymerase gene with outer primers, TGATGCGYTTCTTACCTYTGGTYTCCATGAG and AGTTYTACATGCTGGGCTCTTACCG, and inner primers, GTGACAAAGAGGCTGTCCGTGTCCCCGTA and TCACGTGGCCTACACTTACAAGCCAATCAC, was used as a specific diagnostic for adenoviral shedding.

### Gene finding

We selected 8 genes to use for the subsequent phylogenetic analysis: fiber, hexon, penton base, protease, E1A, DNA-binding protein (DBP), polymerase, terminal protein precursor (pTP). We used the annotated simian adenovirus genome SAdV-25.2 as a reference to find the coding sequences of these genes in the un-annotated (target) genomes. To detect a gene in a target genome, we performed the following procedure: 1) we extracted the focal gene from the reference genome and aligned it to the target genome. This gave us a rough location of the focal gene in the target genome; 2) we detected all ORFs in the target genome that overlapped with the aligned region; 3) if the gene was known to have an intron (E1A, polymerase and pTP and DNA polymerase genes), we detected all GT-AG intron start-stop signal pairs that preserved the length of the first exon up to 30% and the length of the intron up to 60% with respect to the reference gene; all such potential introns were spliced out, generating a pool of potential coding sequences; 4) for each coding sequence from this pool, we aligned the corresponding amino acid sequence to the reference amino acid sequence; 5) we sorted the coding sequences according the their alignment score. The coding sequence with the highest score was considered to be the coding sequence for the focal gene in the target genome. All alignments were performed with the ClustalW v. 2.0.9 software [37].

### Phylogenetic analysis

From the resulting nucleotide sequence alignments for each gene, we reconstructed the phylogenetic trees using the PhyML software [38] under the HKY85 model [39] with a transition/transversion ratio, fraction of invariable sites and the discretized gamma distribution of rates across sites [40]. To assess the reliability of the reconstructed phylogenies, we performed 100 bootstrap reconstructions for each gene.

In order to accurately estimate the evolutionary rates within the B, C, E and D species, we removed all adenoviruses isolated from Old World monkeys as well as HAdV-40 and HAdV-12 isolates. We then aligned the remaining protein sequences and back-translated them to DNA using the PAL2NAL software [41]. We then reconstructed the phylogenies for this subset of data under the same model as before. Finally, we applied the PAML software [42] under the branch-specific model to estimate the dN/dS ratios on different branches keeping the branch lengths fixed. Internal analysis of identity was done by SIMPLOT sliding window analysis (window: 1000 bp, step: 20 bp) [43]. Bootscanning analysis was performed in order to confirm the apparent mosaic pattern of recombination [44].

### Sequence data

In addition to isolates sequenced in this study, the following complete annotated human and simian adenovirus genomes from GenBank were used: NC_010956 (HAdV-9), NC_003266 (HAdV-4), NC_004001 (HAdV-11), NC_001460 (HAdV-12), NC_001405 (HAdV-2), DQ900900 (HAdV-37), AC_000019 (HAdV-35), AC_000017 (HAdV-1), AC_000008 (HAdV-5), NC_001454 (HAdV-40), DQ923122 (HAdV-52), EF153474 (HAdV-26), EF153473 (HAdV-48), DQ393829 (HAdV-49), AY601633 (HAdV-21), AY599836 (HAdV-3), AY594256 (HAdV-7), AY601636 (HAdV-16), AY875648 (HAdV-46), AY737797 (HAdV-34), AY803294 (HAdV-14), AY737798 (HAdV-50), AY771780 (SAdV-1), AY598782 (SAdV-3), CQ982401 (SAdV-6), DQ792570 (SAdV-7), AC000010 (SAdV-21), AY530876 (SAdV-22), AY530877 (SAdV-23), AY530878 (SAdV-24), AC000011 (SAdV-25). All novel adenoviral sequences (SAdV20, SAdV25.2 to SAdV-48) are available in GenBank (FJ025899-FJ025931).

## Supporting Information

Figure S1 Phylogeny of the adenoviral E1a gene. Maximum likelihood analysis under the HKY85 model of substitutions, as described in Materials and Methods and in the legend to Figure 1.
(2.44 MB TIF)

Figure S2 Phylogeny of the adenoviral pre-terminal protein (pTP) gene. Maximum likelihood analysis under the HKY85 model of substitutions, as described in Materials and Methods and in the legend to Figure 1.
(2.46 MB TIF)

Figure S3 Phylogeny of the adenoviral hexon gene. Maximum likelihood analysis under the HKY85 model of substitutions, as described in Materials and Methods and in the legend to Figure 1.
(2.59 MB TIF)

Figure S4 Phylogeny of the adenoviral penton base gene. Maximum likelihood analysis under the HKY85 model of substitutions, as described in Materials and Methods and in the legend to Figure 1.
(2.54 MB TIF)

Figure S5 Phylogeny of the adenoviral protease gene. Maximum likelihood analysis under the HKY85 model of substitutions, as described in Materials and Methods and in the legend to Figure 1.
(2.52 MB TIF)

Figure S6 Phylogeny of the adenoviral DNA-binding protein (DBP) gene. Maximum likelihood analysis under the HKY85 model of substitutions, as described in Materials and Methods and in the legend to Figure 1.
(2.50 MB TIF)

Figure S7 Phylogeny of the adenoviral fiber gene. Maximum likelihood analysis under the HKY85 model of substitutions, as described in Materials and Methods and in the legend to Figure 1.
(2.55 MB TIF)
