# Supplementary figures and images for: Isolation and Characterization of Adenoviruses Persistently Shed from the Gastrointestinal Tract of Non-Human Primates
Source: PLoS Pathog. 2009 Jul 3;5(7):e1000503. doi: 10.1371/journal.ppat.1000503 (PMC2698151; doi:10.1371/journal.ppat.1000503)

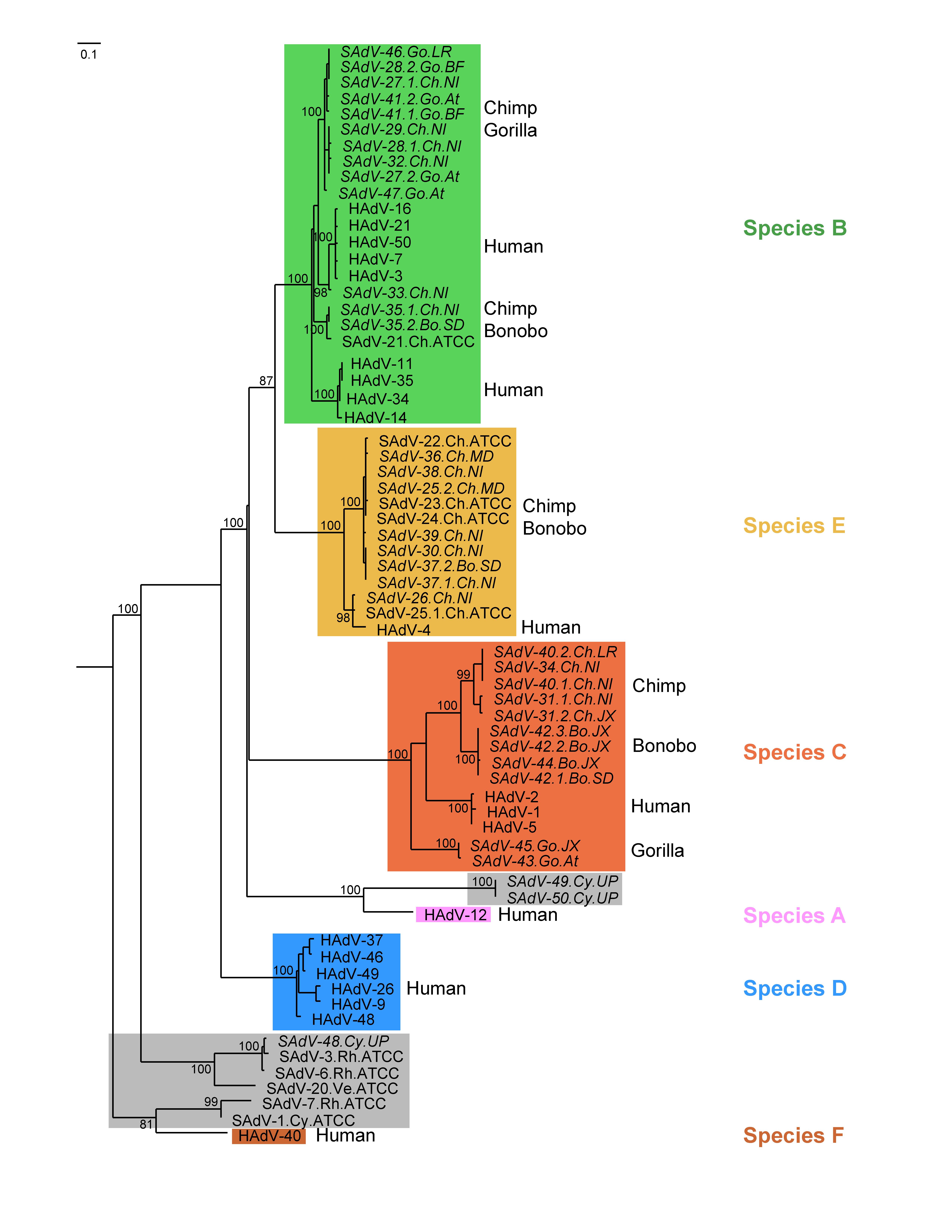

Supplement: Figure S1 — Phylogeny of the adenoviral E1a gene. Maximum likelihood analysis under the HKY85 model of substitutions, as described in Materials and Methods and in the legend to Figure 1. (2.44 MB TIF) [file ppat.1000503.s001.tif]

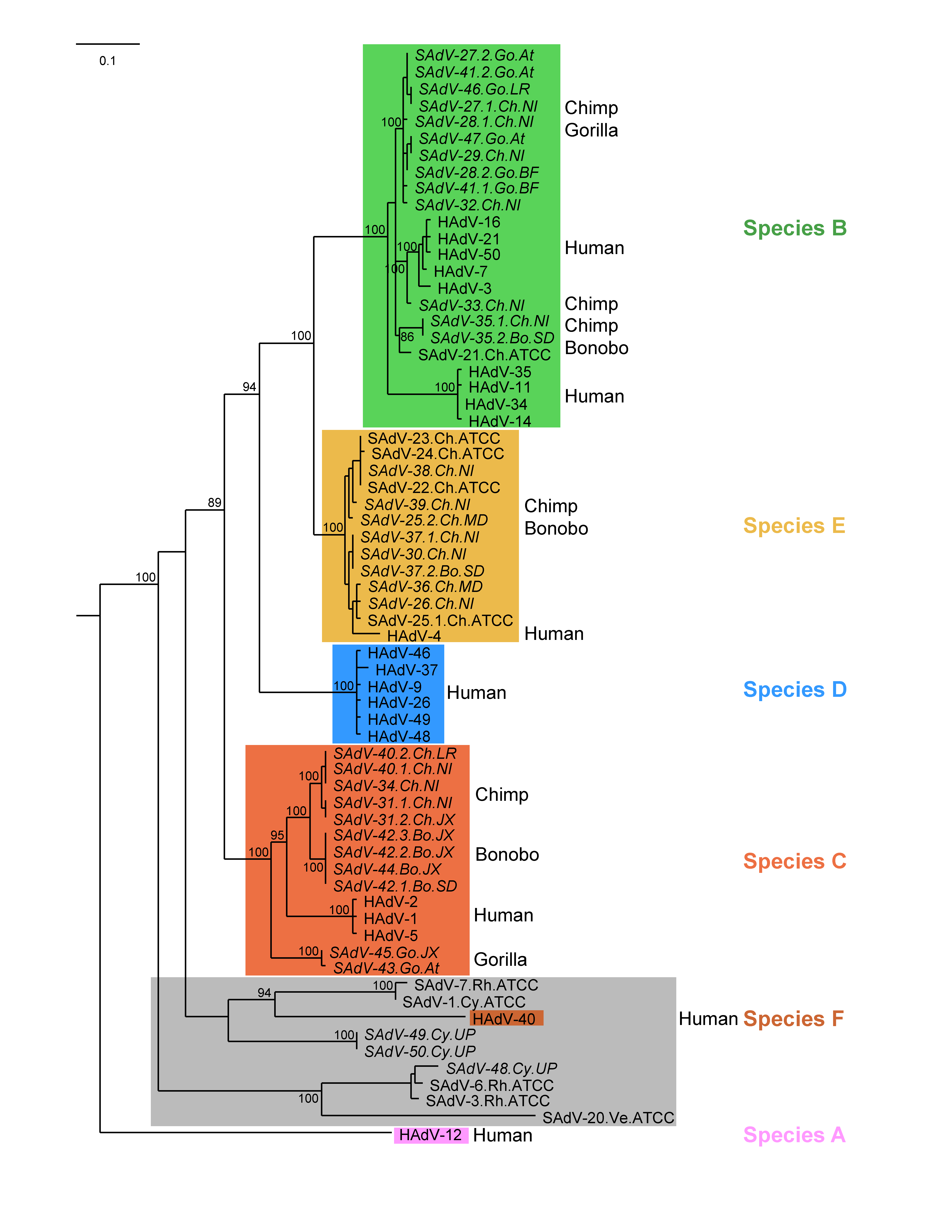

Supplement: Figure S2 — Phylogeny of the adenoviral pre-terminal protein (pTP) gene. Maximum likelihood analysis under the HKY85 model of substitutions, as described in Materials and Methods and in the legend to Figure 1. (2.46 MB TIF) [file ppat.1000503.s002.tif]

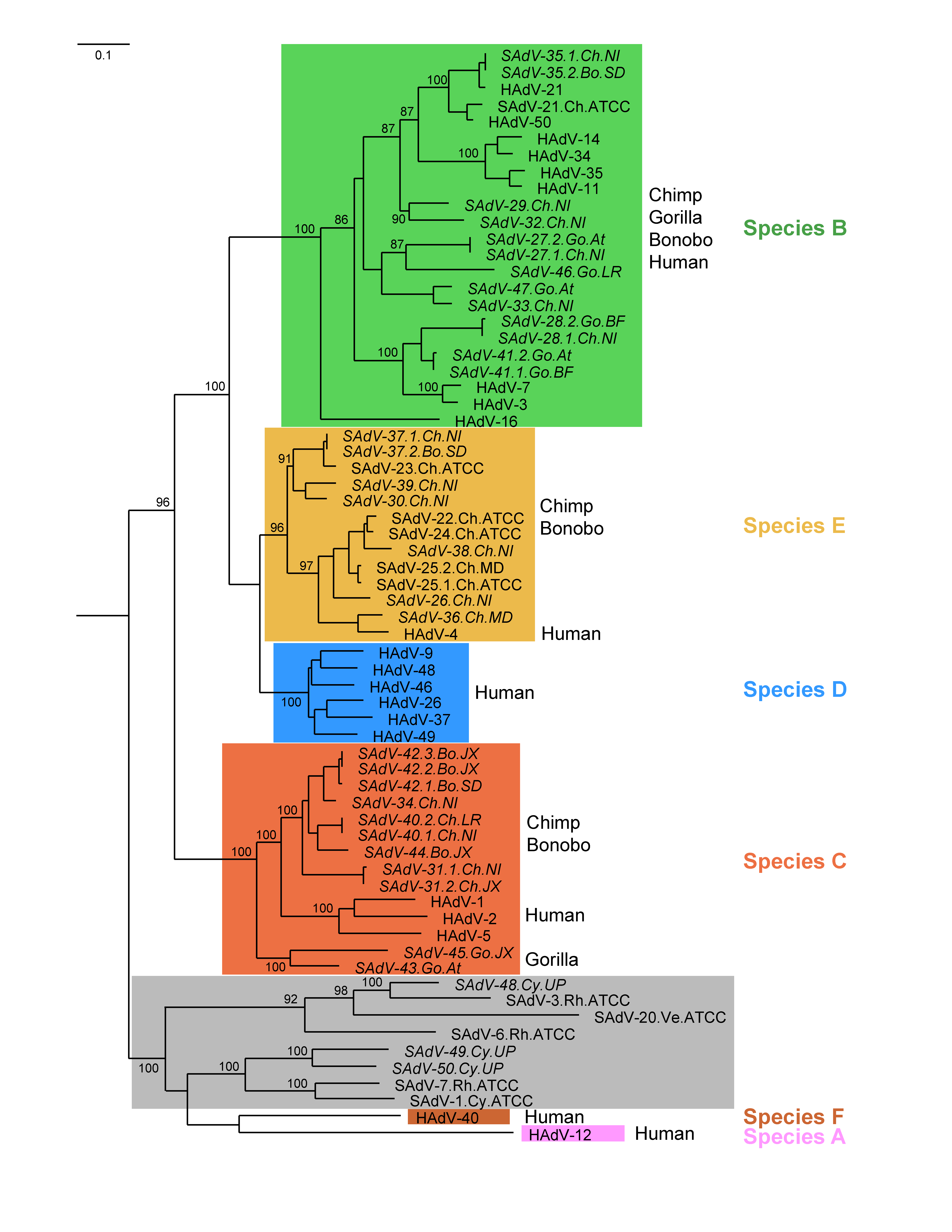

Supplement: Figure S3 — Phylogeny of the adenoviral hexon gene. Maximum likelihood analysis under the HKY85 model of substitutions, as described in Materials and Methods and in the legend to Figure 1. (2.59 MB TIF) [file ppat.1000503.s003.tif]

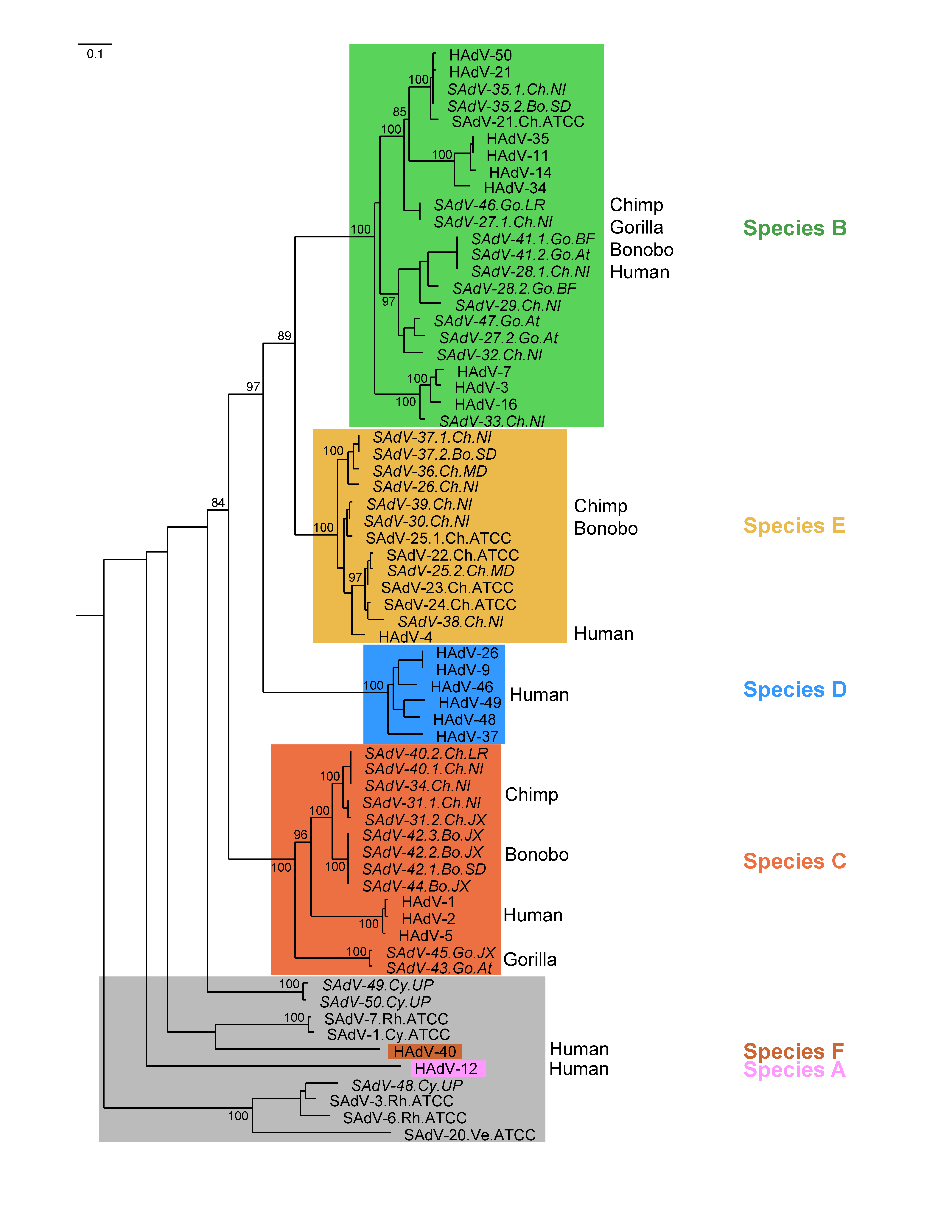

Supplement: Figure S4 — Phylogeny of the adenoviral penton base gene. Maximum likelihood analysis under the HKY85 model of substitutions, as described in Materials and Methods and in the legend to Figure 1. (2.54 MB TIF) [file ppat.1000503.s004.tif]

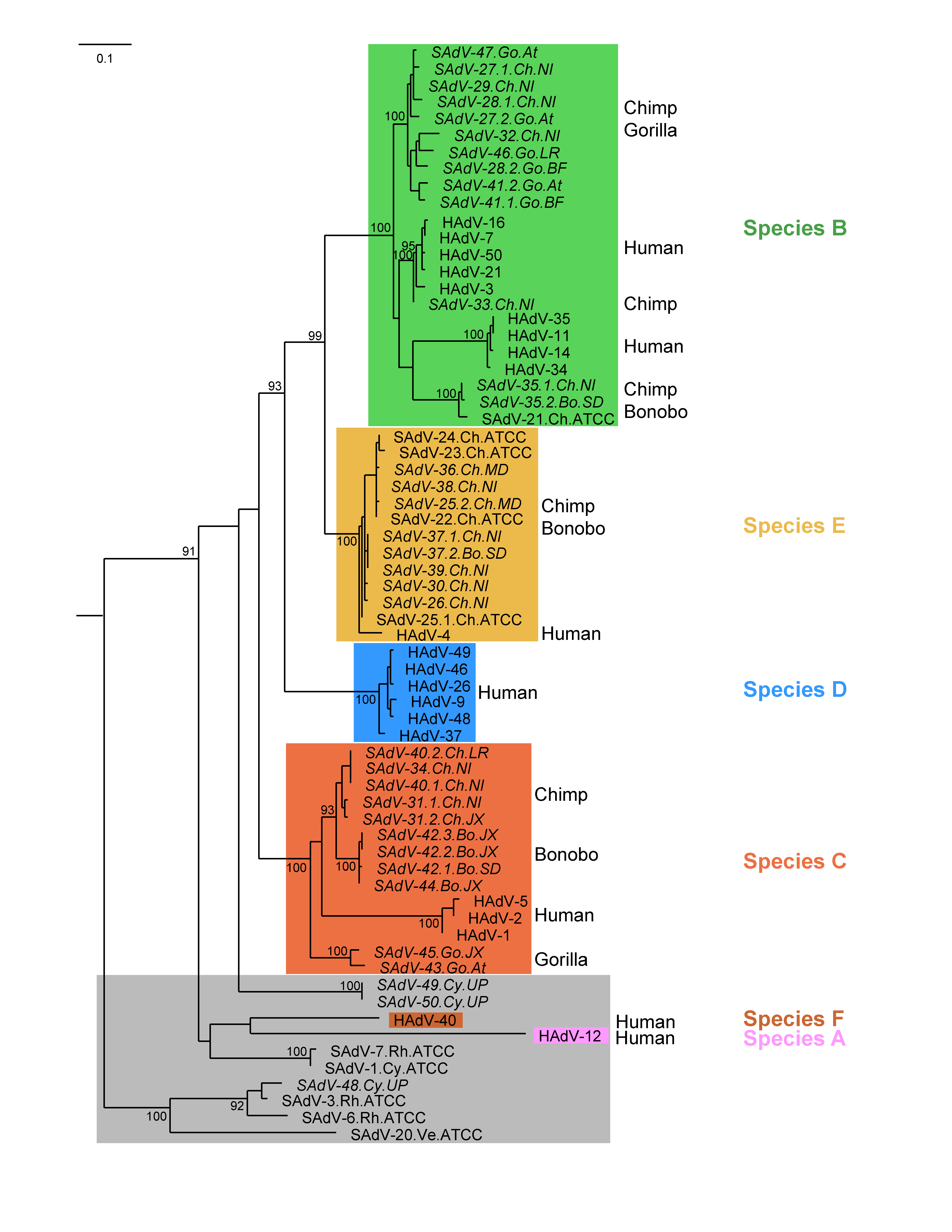

Supplement: Figure S5 — Phylogeny of the adenoviral protease gene. Maximum likelihood analysis under the HKY85 model of substitutions, as described in Materials and Methods and in the legend to Figure 1. (2.52 MB TIF) [file ppat.1000503.s005.tif]

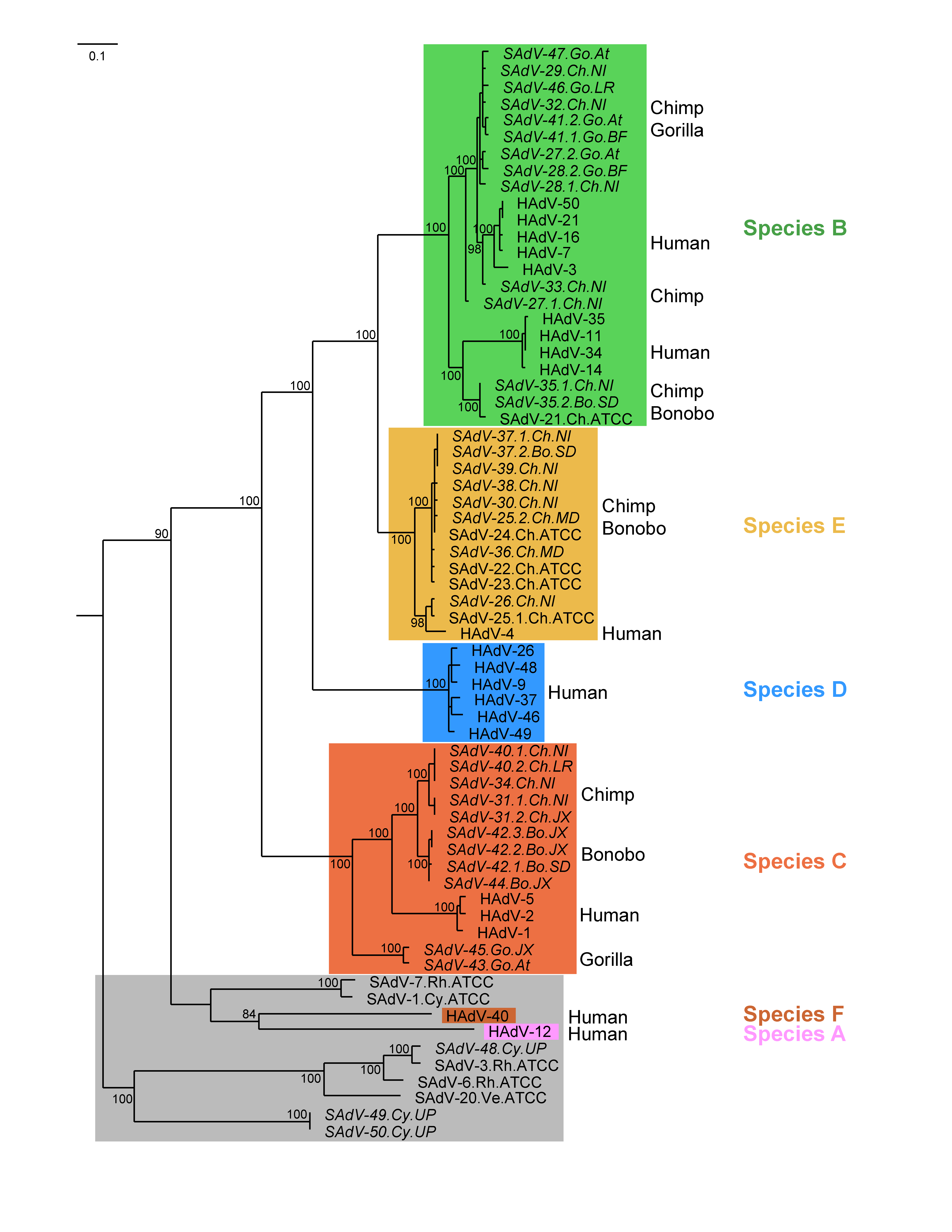

Supplement: Figure S6 — Phylogeny of the adenoviral DNA-binding protein (DBP) gene. Maximum likelihood analysis under the HKY85 model of substitutions, as described in Materials and Methods and in the legend to Figure 1. (2.50 MB TIF) [file ppat.1000503.s006.tif]

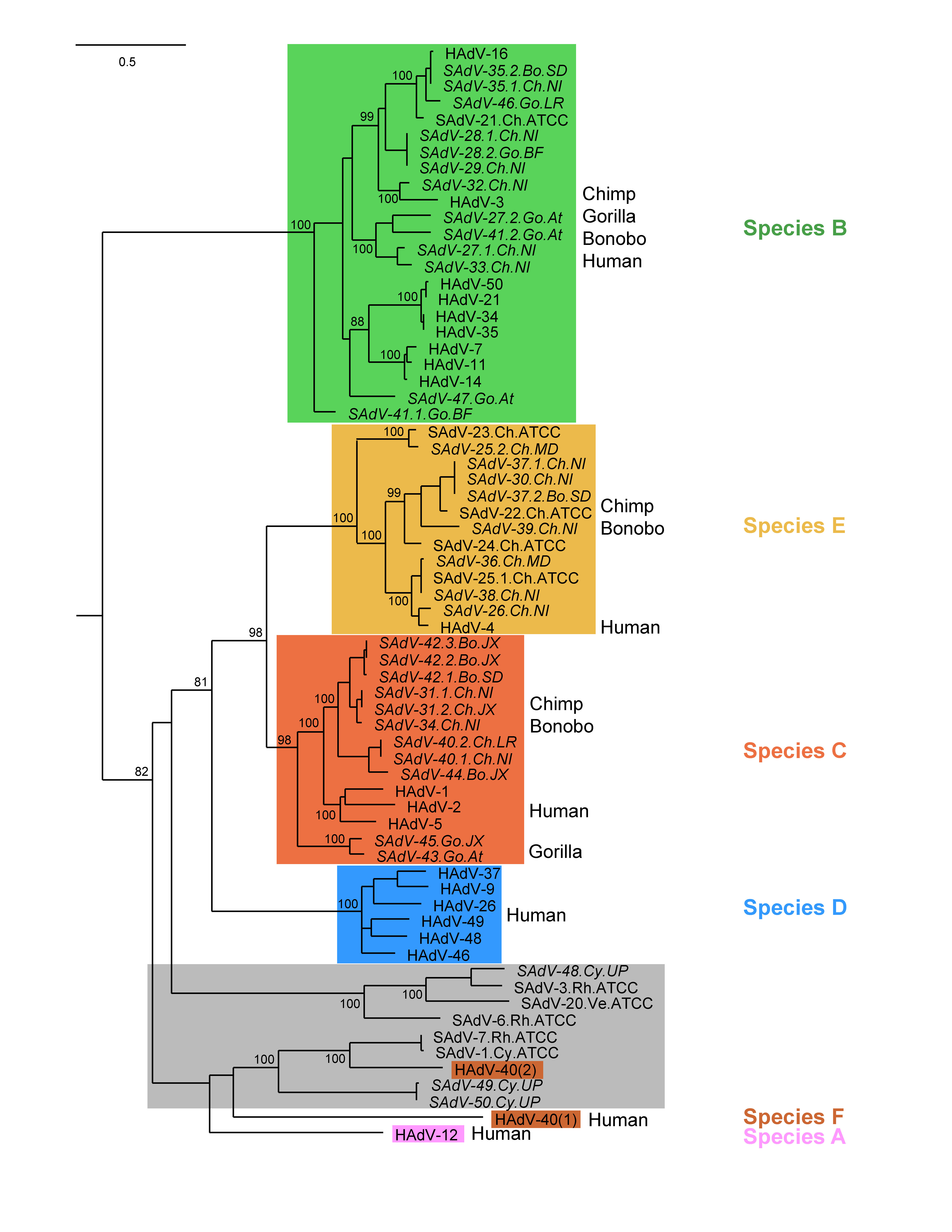

Supplement: Figure S7 — Phylogeny of the adenoviral fiber gene. Maximum likelihood analysis under the HKY85 model of substitutions, as described in Materials and Methods and in the legend to Figure 1. (2.55 MB TIF) [file ppat.1000503.s007.tif]
